# Targeted therapy using polymyxin B hemadsorption in patients with sepsis: a post-hoc analysis of the JSEPTIC-DIC study and the EUPHRATES trial

**DOI:** 10.1186/s13054-023-04533-3

**Published:** 2023-06-21

**Authors:** Itsuki Osawa, Tadahiro Goto, Daisuke Kudo, Mineji Hayakawa, Kazuma Yamakawa, Shigeki Kushimoto, Debra M. Foster, John A. Kellum, Kent Doi

**Affiliations:** 1 Department of Emergency and Critical Care Medicine, The University of Tokyo Hospital, 7-3-1, Hongo, Bunkyo-Ku, Tokyo 1130033 Japan; 2 Department of Clinical Epidemiology and Health Economics, School of Public Health, The University of Tokyo, Tokyo, Japan; 3 TXP Medical Co. Ltd., Tokyo, Japan; 4 Division of Emergency and Critical Care Medicine, Tohoku University Graduate School of Medicine, Miyagi, Japan; 5 Department of Emergency Medicine, Hokkaido University Hospital, Hokkaido, Japan; 6 Department of Emergency and Critical Care Medicine, Osaka Medical and Pharmaceutical University, Osaka, Japan; 7 Spectral Medical, Toronto, ON Canada; 8 Department of Critical Care Medicine, Center for Critical Care Nephrology, University of Pittsburgh, Pittsburgh, PA USA

**Keywords:** Endotoxemia, Endotoxin, Hemadsorption, Polymyxin B, Sepsis

## Abstract

**Background:**

Polymyxin B hemadsorption (PMX-HA) reduces blood endotoxin levels, but characteristics of patients with sepsis likely to benefit from PMX-HA are not well known. We sought to identify patient subgroups likely to benefit from PMX-HA.

**Methods:**

We retrospectively identified 1911 patients with sepsis from a retrospective observational study in Japan (the JSEPTIC-DIC study) and 286 patients with endotoxemic septic shock from a randomized controlled trial in North America that restricted patients to those with high endotoxin activity (the EUPHRATES trial). We applied the machine learning-based causal forest model to the JSEPTIC-DIC cohort to investigate heterogeneity in treatment effects of PMX-HA on 28-day survival after adjusting for potential confounders and ascertain the best criteria for PMX-HA use. The derived criteria for targeted therapy by PMX-HA were validated using the EUPHRATES trial cohort.

**Results:**

The causal forest model revealed heterogeneity in treatment effects of PMX-HA. Since patients having higher treatment effects were more likely to have severe coagulopathy and hyperlactatemia, we identified the potential treatment targets of PMX-HA as patients with PT-INR > 1.4 or lactate > 3 mmol/L. In the EUPHRATES trial cohort, PMX-HA use on the targeted subpopulation (75% of all patients) was significantly associated with higher 28-day survival (PMX-HA vs. control, 68% vs. 52%; treatment effect of PMX-HA, + 16% [95% CI + 2.2% to + 30%], *p* = 0.02).

**Conclusions:**

Abnormal coagulation and hyperlactatemia in septic patients with high endotoxin activity appear to be helpful to identify patients who may benefit most from PMX-HA. Our findings will inform enrollment criteria for future interventional trials targeting patients with coagulopathy and hyperlactatemia.

**Supplementary Information:**

The online version contains supplementary material available at 10.1186/s13054-023-04533-3.

## Background

Endotoxin, a part of the outer membrane of gram-negative bacteria, can trigger both a host immune response and multiple organ failure. Higher endotoxin activity is associated with greater degrees of organ failure and higher mortality in sepsis [1, 2]. To prevent the progression of organ failure caused by endotoxemia, clinicians have used Polymyxin B hemadsorption (PMX-HA), formerly known as Polymyxin B direct hemoperfusion (PMX-DHP), an extracorporeal blood purification modality to remove circulating endotoxin by adsorption [3, 4]. Several studies have suggested that PMX-HA improves clinical outcomes in patients with sepsis [5, 6]; however, one of the randomized controlled trials (RCTs), the ABDOMIX trial, did not show clinical benefit of PMX-HA use in patients with peritonitis-induced septic shock [7].

The EUPHRATES trial, another RCT using PMX-HA, enrolled only patients with septic shock and higher levels of endotoxin activity (Endotoxin Assay Activity [EAA] ≥ 0.60), who were considered potential responders of PMX-HA, but failed to show a significant reduction in 28-day mortality [8]. One possible reason for this result was the target range of EAA in the EUPHRATES trial was not optimal [9]. Indeed, a post-hoc analysis of the EUPHRATES trial reported that two PMX-HA treatments could significantly reduce mortality in patients with septic shock and EAA between 0.60 and 0.90 [10]. In addition to this, given that previous studies showed PMX could be effective to improve survival in septic patients with significant organ failures [8, 11], the effect of PMX-HA may be heterogeneous across patient phenotypes.

In this context, we sought to identify previously unidentified patient subgroups with sepsis who may benefit from targeted therapy using PMX-HA. For this study, the derived criteria for targeted use of PMX-HA were determined with the machine learning-based causal forest model in an observational cohort (the JSEPTIC-DIC cohort) and validated in an RCT cohort (the EUPHRATES trial cohort).

## Methods

### Study design and data

The concept of this study is shown in Additional file 1: Fig. S1. This is a secondary analysis of the Japan Septic Disseminated Intravascular Coagulation (JSEPTIC-DIC) study cohort (UMIN-CTR ID: UNIN000012543) [12] and the EUPHRATES trial cohort (Clinicaltrials.gov ID: NCT01046669) [8]. The JSEPTIC-DIC study retrospectively collected data on 3195 adult patients (aged ≥ 16 years) with severe sepsis or septic shock based on the International Sepsis Definitions Conference criteria [13], who were admitted to 40 tertiary hospitals (42 ICUs) in Japan, between January 2011 and December 2013. Another study using this dataset reported a possible treatment effect of PMX-HA estimated by propensity score matching [14]. The EUPHRATES trial was an RCT that enrolled 450 adult patients (aged ≥ 18 years) with septic shock and EAA ≥ 0.60 who were admitted to 55 tertiary hospitals in the USA and Canada, between September 2010 and June 2016. The study protocol entitled “Targeted therapy using Polymyxin B hemadsorption in patients with sepsis” was approved by the Institutional Review Board of the University of Tokyo Hospital (No. 2022332NI) on February 27, 2023. This study was a secondary data analysis of de-identified data, and therefore, the requirement for informed consent was waived.

**Fig. 1:**
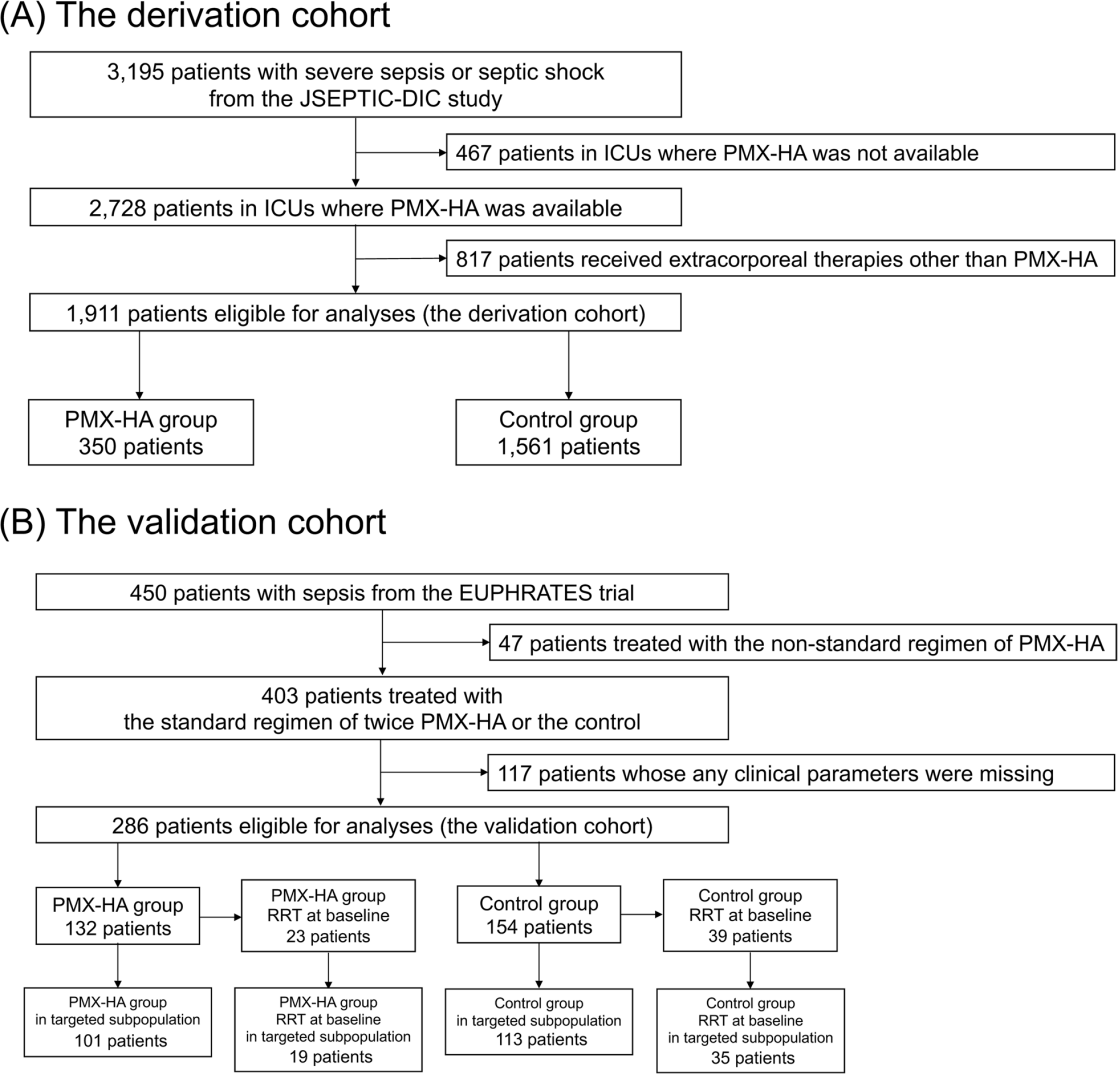
Flow diagram of patients eligible for analysis in the derivation and validation cohorts. Legend: **A** Among 3195 patients with severe sepsis or septic shock based on the International Sepsis Definitions Conference criteria from the JSEPTIC-DIC study, 2011–2013, we identified 1911 patients with sepsis who did not receive extracorporeal therapies other than PMX-HA as the derivation cohort. **B** Among 450 patients with septic shock from the EUPHRATES trial, 2010–2016, we identified 286 patients who received the standard regimen of PMX-HA treatments or standard of care without PMX-HA who had complete data available (i.e., APACHE II score, SOFA score, WBC count, platelet count, PT-INR, and lactate). PMX-HA = Polymyxin B Hemadsorption, RRT = Renal Replacement Therapy, APACHE II score = Acute Physiology and Chronic Health Enquiry II score, SOFA score = Sequential Organ Failure Assessment score, WBC = White Blood Cell, PT-INR = Prothrombin Time and International Normalized Ratio

### Study participants

Among 3195 patients from the JSEPTIC-DIC study cohort, we identified 1911 patients with severe sepsis or septic shock who were admitted 34 ICUs where PMX-HA was available. High prevalence of patients on renal replacement therapy (RRT) among those subsequently treated with PMX-HA was reported by previous studies possibly because of easier access to extracorporeal circulation [14, 15]. We attempted to exclude patients who received RRT right before PMX-HA to address the potential bias due to the high propensity of PMX-HA initiation to patients on RRT. However, given the absence of data on timing of initiation of RRT and PMX-HA, we excluded all patients treated with RRT when analyzing the JSEPTIC-DIC study cohort.

Among 450 patients from the EUPHRATES trial cohort, we identified 286 patients treated with the standard regimen of PMX-HA treatments or standard of care without PMX-HA who had complete data available for Acute Physiology And Chronic Health Evaluation II (APACHE II) score, Sequential Organ Failure Assessment (SOFA) score, white blood cell (WBC) count, platelet count, prothrombin time-international normalized ratio (PT-INR), and blood lactate concentration.

We used the JSEPTIC-DIC study cohort as a derivation cohort to identify targeted subpopulations of PMX-HA and EUPHRATES trial cohort as a validation cohort to verify the derived criteria, respectively (Fig. 1).

### Measurements

We used the following parameters on the first day of ICU admission (i.e., before initiation of specific treatment for sepsis [e.g., PMX-HA and anticoagulants]) for analysis in the derivation cohort: patient demographics (i.e., age and sex), APACHE II score, SOFA score and each component of the SOFA score, WBC count, platelet count, PT-INR, fibrinogen, fibrinogen/fibrin degradation products (FDP) and lactate level. Missing variables were imputed using the random forest method, a nonparametric algorithm that can accommodate nonlinearities and interactions and does not require a particular parametric model to be specified [16]. All missing data were imputed using all parameters shown in Additional file 1: Table S1 and 28-day survival.

**Table 1:** Patient characteristics in each quintile in order of estimated individual treatment effects, derivation cohort

Characteristic	Quintile 1 (*n* = 390)	Quintile 2 (*n* = 380)	Quintile 3 (*n* = 380)	Quintile 4 (*n* = 380)	Quintile 5 (*n* = 381)
	Lower treatment effects				Higher treatment effects
*Patient demographics*					
Age*	64 (53–69)	68 (58–76)	76 (70–82)	78 (71–84)	79 (71–84)
Female sex, *n* (%)*	156 (40%)	164 (43%)	168 (44%)	164 (43%)	165 (43%)
*Infection site, n (%)*					
Lung	99 (25%)	101 (27%)	91 (24%)	99 (26%)	99 (26%)
Abdominal	104 (27%)	110 (29%)	126 (33%)	159 (42%)	143 (38%)
Blood culture positive, *n* (%)	288 (74%)	272 (72%)	304 (80%)	282 (74%)	284 (75%)
*Patient severity*					
APACHE II score*	19 (14–25)	18 (14–23)	19 (15–23)	21 (17–25)	30 (23–37)
SOFA score*	8 (5–10)	7 (5–9)	7 (5–9)	8 (6–11)	12 (9–14)
Respiratory*	2 (1–3)	2 (1–3)	2 (1–3)	2 (1–3)	3 (2–3)
Coagulation*	0 (0–2)	0 (0–2)	1 (0–2)	1 (0–2)	1 (0–2)
Liver*	0 (0–1)	0 (0–1)	0 (0–1)	0 (0–1)	0 (0–1)
Cardiovascular*	1 (0–3)	2 (0–3)	2 (0–3)	2 (0–3)	3 (2–4)
Central nervous system*	1 (0–3)	1 (0–2)	1 (0–2)	1 (0–2)	3 (1–4)
Renal*	1 (0–2)	0 (0–1)	0 (0–1)	1 (0–2)	2 (1–3)
*Laboratory tests*					
White blood cells [× 10^3^/μL]*	12 (6–22)	12 (6–19)	12 (6–17)	11 (5–16)	8 (3–16)
Platelet [× 10^4^/μL]*	15.0 (8.1–23.5)	14.9 (9.1–22.3)	14.0 (9.0–20.8)	13.8 (8.0–20.6)	10.3 (5.3–17.2)
PT-INR*	1.28 (1.13–1.44)	1.26 (1.12–1.42)	1.27 (1.15–1.46)	1.33 (1.18–1.51)	1.50 (1.29–1.85)
Fibrinogen [mg/dL]*	487 (366–650)	441 (346–553)	431 (341–524)	406 (277–499)	310 (185–426)
FDP [μg/mL]*	22 (12–33)	20 (11–29)	20 (11–31)	23 (14–40)	36 (18–66)
Lactate [mmol/L]*	2.3 (1.5–3.3)	2.3 (1.5–3.3)	2.3 (1.6–3.5)	4.1 (2.2–5.9)	5.9 (4.4–8.2)
Treatment effects of PMX-HA on 28-day survival [95% CI]	− 4.9% [− 24% to + 14%]	− 0.0% [− 9.3% to + 9.2%]	+ 1.8% [− 6.5% to + 10%]	+ 8.8% [+ 0.1% to + 18%]	+ 21% [+ 11% to + 32%]

Values represent median (IQR), unless otherwise indicated. Higher treatment effects on 28-day survival are equivalent to higher absolute risk reduction of 28-day mortality

APACHE II score, Acute Physiology and Chronic Health Enquiry II score; SOFA score, Sequential Organ Failure Assessment score; PT-INR, Prothrombin Time and International Normalized Ratio; FDP, Fibrinogen/Fibrin Degradation Products; PMX-HA, Polymyxin B Hemadsorption

*Variables were used for adjusting the causal forest model

### Exposures

The exposures were at least one PMX-HA treatment in the derivation cohort and the standard regimen of two PMX-HA treatments in the validation cohort (i.e., per-protocol analysis of the EUPHRATES trial).

### Outcome measures

The main outcome was 28-day survival in both the derivation and validation cohorts. In the validation cohort, the secondary outcome was survival time within 28 days of follow-up period. We also estimated the treatment effects of PMX-HA on 28-day survival without any RRT (i.e., survival 28 days after ICU admission without ever receiving RRT) because the analysis was limited to patients who were treated without RRT in the derivation cohort.

### Statistical analysis

We first computed summary statistics to delineate baseline patient characteristics at ICU admission and compare the characteristics between patients treated with and without PMX-HA both in the derivation and validation cohort.

Next, to evaluate the heterogeneity in the treatment effects of PMX-HA, we applied the machine learning-based causal forest model to the derivation cohort using the *grf* package in R [17–19]. The causal forest model is a machine learning-based model for causal inference developed in the field of econometrics to estimate treatment effects at the individual level and to detect treatment effect heterogeneity in high-dimensional settings. Our causal forest model was constructed from 5000 causal trees with tenfold cross-fitting in addition to the “honest” approach to minimize bias due to overfitting [18, 20], where a quarter of the data was used to construct the tree structure, another quarter of the data was used to make predictions, and the remaining data were used to test the developing model [20, 21]. All hyperparameters in the causal forest algorithm were tuned by cross-validation [17]. The model calibration was assessed by an estimate of the best linear predictor of true conditional average treatment effects based on out-of-bag predictions [17, 18, 21]. The coefficient of the mean causal forest prediction close to one indicates a well-calibrated model, and a large, statistically significant coefficient of the out-of-bag predictions suggest that the causal forest captured heterogeneity. More details on causal forest analysis have been shown in previous studies [18–22].

Based on the constructed causal forest model, we estimated the individual treatment effects (ITEs) of PMX-HA on 28-day survival in all patients from the derivation cohort and ranked them into quintiles of estimated ITEs. We estimated conditional average treatment effects in each quintile group to evaluate the heterogeneity in treatment effects of PMX-HA and compared the characteristics in each group in order to identify the candidate determinants of treatment effects. To investigate the contribution of the candidate determinants to the variation in the treatment effects of PMX-HA, we drew heatmaps of predicted treatment effects, conditioning on the candidate determinants with the rest of the covariates fixed at the median for continuous variables and at the mode for binary variables [18]. By applying the policytree algorithm (*policytree* package in R) to the estimated ITEs in the derivation cohort, we statistically chose the best criteria for PMX-HA initiation (e.g., the optimal combination of parameters and their corresponding thresholds) to maximize the benefit of the treatment effect in the entire population using the heuristically-selected candidate determinants of PMX-HA treatment effects among all measurements [23, 24]. The treatment effects of PMX-HA in the full sample and the targeted population of the derivation cohort were estimated using the augmented inverse probability weighting formula with our causal forest model [25].

Finally, to validate the criteria derived from our causal forest model, we estimated treatment effects of PMX-HA on 28-day survival in the (1) full sample and (2) targeted subpopulation of the validation cohort (i.e., patients who met the derived criteria). We also performed survival analysis for up to 28 days using Kaplan–Meier curves with the log-rank test and the Cox proportional hazard model adjusted for APACHE II and SOFA scores.

To address the bias due to the exclusion of all patients who were treated with RRT from the derivation cohort, we estimated treatment effects of PMX-HA on 28-day survival in patients who were on RRT and not on RRT at randomization for PMX-HA in the targeted subpopulation of the validation cohort.

A *P*-value of < 0.05 was considered statistically significant. We performed all analyses with R (version 4.1.1). We have deposited our analysis code using the derivation cohort to a public repository (https://github.com/iosawa/HTE_PMX-HA).

## Results

### Participants in the derivation and validation cohorts

Among 1911 patients in the derivation cohort eligible for analysis, 350 received PMX-HA (median age, 72 years; women, 164 [47%]; median APACHE II score, 21; median SOFA score, 9) and 1561 did not receive PMX-HA (median age, 74 years; women, 653 [42%]; median APACHE II score, 21; median SOFA score, 8) (Additional file 1: Table S2). Of these, the 28-day survival rate in the PMX-HA group was 85% (297 of 350) compared with 82% (1275 of 1561) in the control group.

**Table 2:** Estimated treatment effects of Polymyxin B hemadsorption in the derivation and validation cohorts

	Treatment effects on 28-day survival [95% CI]	Hazard ratio for 28-day mortality [95% CI]	Treatment effects on 28-day survival without RRT initiation [95% CI]
*Derivation cohort*			
Full sample (*n* = 1911)	+ 5.3% [− 1.9% to + 13%]	–	–
Targeted subpopulation (*n* = 1203)	+ 9.2% [+ 1.0% to + 17%]	–	–
Full sample (*n* = 286)	+ 7.4% [− 4.3% to + 19%], (PMX-HA vs. control, 70% vs. 62%)	0.71 [0.48 to 1.06]	+ 2.9% [− 9.0% to + 15%] (PMX-HA vs. control, 39% vs. 36%)
*Validation cohort*			
Targeted subpopulation (*n* = 214)	+ 16% [+ 2.2% to + 30%] (PMX-HA vs. control, 68% vs. 52%)	0.62 [0.40 to 0.95]	+ 12% [− 1.4% to + 25%] (PMX-HA vs. control, 37% vs. 25%)
Patients on RRT at baseline (*n* = 54)	+ 7.4% [− 24% to + 39%] (PMX-HA vs. control, 47% vs. 40%)	0.56 [0.25 to 1.27]	–
Patients not on RRT at baseline (*n* = 160)	+ 15% [− 0.3% to + 31%] (PMX-HA vs. control, 73% vs. 58%)	0.68 [0.40 to 1.16]	+ 11% [− 5.8% to + 27%] (PMX-HA vs. control, 45% vs. 35%)

Higher treatment effects on 28-day survival are equivalent to higher absolute risk reduction of 28-day mortality. “Targeted subpopulation” indicates patients with PT-INR > 1.4 or lactate > 3 mmol/L on ICU admission. The estimate of each treatment effect of PMX-HA in the derivation cohort are computed using the augmented inverse probability weighting formula with the causal forest model. The estimated treatment effects in the validation cohort were calculated using the per-protocol analysis utilizing the nature of a RCT

RRT, Renal Replacement Therapy; PT-INR, Prothrombin Time and International Normalized Ratio; PMX-HA, Polymyxin B Hemadsorption; RCT, Randomized Controlled Trial

Among 286 patients in the validation cohort eligible for analysis, 132 received PMX-HA in addition to standard of care (median age, 62 years; women, 54 [41%]; median APACHE II score, 30; median SOFA score, 12; median EAA, 0.79) and 154 received the standard of care without PMX-HA (median age, 58 years; women, 61 [40%]; median APACHE II score, 29; median SOFA score, 12; median EAA, 0.75) (Additional file 1: Table S3). Of these, the 28-day survival rate in the PMX-HA group was 70% (92 of 132) compared with 62% (96 of 154) in the control group. 22% (62 of 286) in this cohort were on RRT at randomization.

### Development of causal forest model in the derivation cohort

**Fig. 2:**
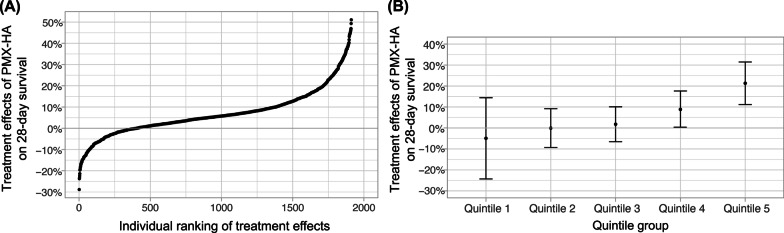
Distribution of estimated individual treatment effects and conditional average treatment effects within each quintile of estimated individual treatment effects. Shown are **A** the distribution of estimated individual treatment effects of PMX-HA on 28-day survival and **B** the conditional average treatment effects of PMX-HA within each quintile of estimated individual treatment effects. Higher treatment effects on 28-day survival are equivalent to higher absolute risk reduction of 28-day mortality. Both graphs visually point to the presence of heterogeneity in the effects of PMX-HA on 28-day survival in patients with sepsis. PMX-HA = Polymyxin B Hemadsorption

As shown in Fig. 2A indicating all individual treatment effects, our causal forest model suggested heterogeneity in treatment effects of PMX-HA on 28-day survival. Treatment effects of PMX-HA among individuals in the top 20% of the estimated individual treatment effects were significantly higher than that of others (treatment effects on 28-day survival in the top 20% vs. in the remaining 80%, + 21% [95% CI + 7.5% to + 35%] vs. + 1.4% [95% CI − 6.1% to + 8.9%]) (Fig. 2B). In the best linear predictor of true conditional average treatment effects, the coefficient of the mean causal forest prediction was 0.94 (*p* = 0.045), indicating that the mean causal forest prediction was well calibrated. In addition, the coefficient of the out-of-bag predictions was 0.66 (*p* = 0.01), suggesting that the forest captured heterogeneity [17, 18, 21].

### Potential targets of Polymyxin-B hemadsorption based on the derivation cohort

By comparing the characteristics in each quintile group based on the estimated individual treatment effects, we found that patient severity scores (i.e., APACHE II and SOFA scores), parameters indicating coagulopathy (e.g., platelet, PT-INR, and FDP), and lactate could be the key determinants of treatment effects of PMX-HA (Table 1). Therefore, based on the relationship between each parameter and individual treatment effects of PMX-HA, we heuristically selected four factors, APACHE II score, platelet, PT-INR, and lactate, as the candidate determinants of treatment effects of PMX-HA. We also showed how much these factors contributed to the values of PMX-HA treatment effects using heatmaps (Additional file 1: Fig. S2). The treatment effects of PMX-HA are defined by the variation of multiple parameters (e.g., patients with the same lactate level but higher PT-INR are likely to benefit from PMX-HA). The policytree algorithm statistically selected patients with [PT-INR > 1.43] or [PT-INR <  = 1.43 and lactate > 3.22 mmol/L] as optimal targets of PMX-HA. For simplicity, we identified patients with PT-INR > 1.4 or lactate > 3 mmol/L as “targeted subpopulation” in this study (Additional file 1: Fig. S3).

**Fig. 3:**
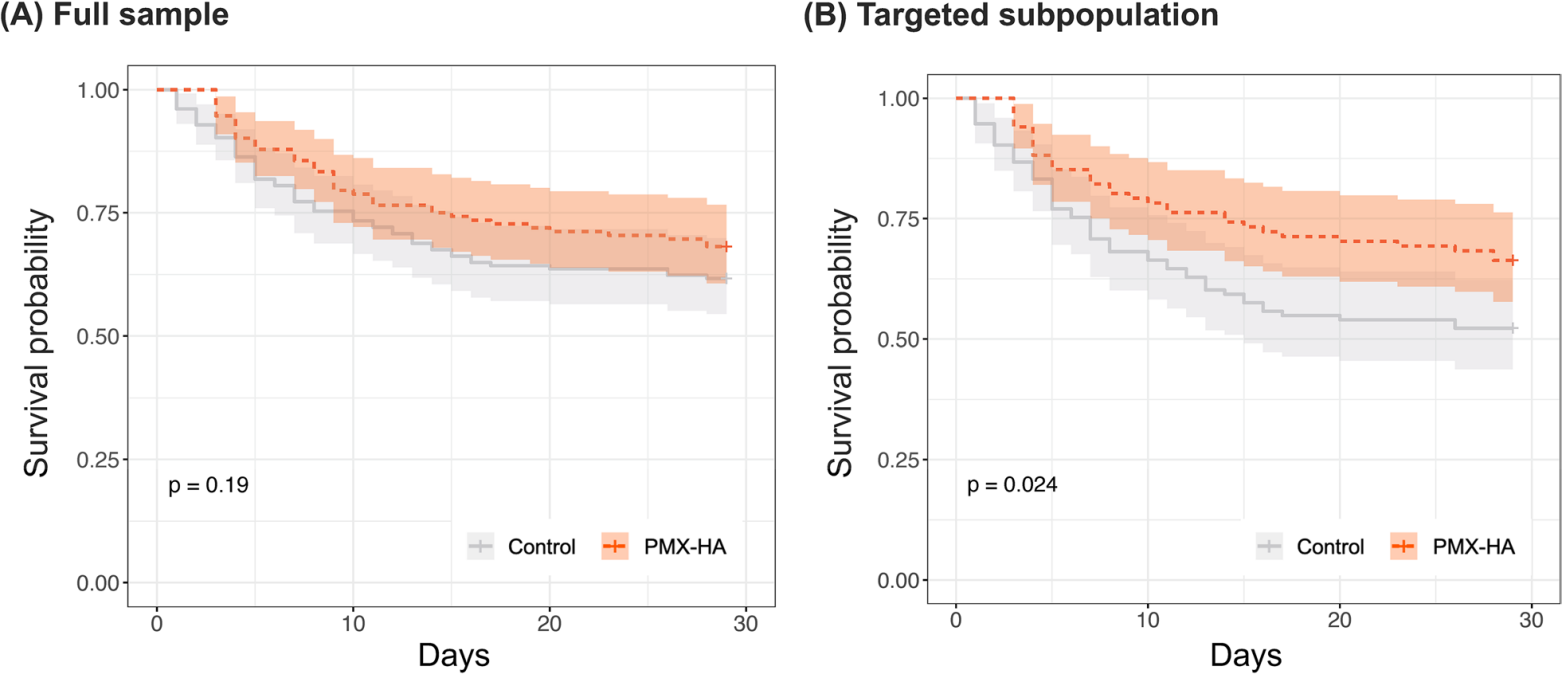
Kaplan–Meier curves of patients in all and targeted population of the validation cohort. Legend: Shown are unadjusted Kapan–Meier curves of patients in **A** all and **B** targeted population of the validation cohort. “Targeted subpopulation” indicates patients with PT-INR > 1.4 or lactate > 3 mmol/L on ICU admission. P values were estimated by the log-rank test. The hazard ratios up to 28 days using the Cox proportional hazard model, adjusted for baseline APACHE II and SOFA scores, are 0.71 (95% CI [0.48 to 1.06], *p* = 0.10) and 0.62 (95% CI [0.40 to 0.95], *p* = 0.03) in all and targeted population, respectively. PMX-HA = Polymyxin B Hemadsorption, PT-INR = Prothrombin Time and International Normalized Ratio, APACHE II score = Acute Physiology and Chronic Health Enquiry II score, SOFA score = Sequential Organ Failure Assessment

PMX-HA use on the targeted subpopulation of the derivation cohort had statistically significant higher treatment effects on 28-day survival (+ 9.2% [95% CI + 1.0% to + 17%], *p* < 0.01). (Table 2) The targeted subpopulation accounted for 63% (1203 out of 1911) and 75% (214 out of 286) of the derivation and validation cohort, respectively. The characteristics of the targeted population in the derivation and validation cohort are shown in Additional file 1: Tables S4 and S5.

### Verification of our criteria in the validation cohort

In the validation cohort, PMX-HA use in the full sample did not significantly improve 28-day survival (28-day survival rate in the PMX-HA group vs. in the control group, 70% vs. 62%, + 7.4% [95% CI − 4.3% to + 19%], *p* = 0.20), but PMX-HA use in the targeted subpopulation significantly improved survival (68% vs. 52%, + 16% [95% CI + 2.2% to + 30%], *p* = 0.02) (Table 2). Similar associations were observed in the survival analysis, as shown in the Kaplan–Meier curves (Fig. 3).

As for the secondary outcomes, using the Cox proportional hazard model, we found a significant difference in the adjusted survival time to 28 days between PMX-HA versus control in the targeted population of the validation cohort (hazard ratio [HR] 0.62, 95% CI 0.40 to 0.95, *p* = 0.03), but no significant difference in the full sample of the validation cohort (HR 0.71, 95% CI 0.48 to 1.06, *p* = 0.10) (Table 2 and Fig. 3).

### Additional analysis in the validation cohort

The treatment effects of PMX-HA on 28-day survival among patients in the targeted subpopulation not on RRT at baseline (PMX-HA vs. control, 73% vs. 58%, + 15% [95% CI − 0.3% to + 31%], *p* = 0.058; Table 2) were comparable to the treatment effect on the full targeted subpopulation including both those on RRT and not on RRT at baseline (68% vs. 52%, + 16% [95% CI + 2.2% to + 30%], *p* = 0.02). However, for the targeted population on RRT at baseline, there was a statistically non-significant treatment effect in favor of PMX-HA (PMX-HA vs. control, 47% vs. 40%, + 7.4% [95% CI − 39% to + 24%], *p* = 0.80). Using an adjusted survival time to 28 days using the Cox proportional hazard model among patients in the targeted population on RRT at baseline, the benefit for PMX-HA was mathematically improved but remained non-statistically significant (HR 0.56, 95% CI 0.25 to 1.27, *p* = 0.17), as was similarly seen in the adjusted survival time among patients in the targeted subpopulation not on RRT at baseline (HR 0.68, 95% CI 0.40 to 1.16, *p* = 0.16).

In addition, PMX-HA use was likely to have higher effects on 28-day survival without RRT initiation in the targeted subpopulation (28-day survival rate without RRT initiation in the PMX-HA group vs. control, 37% vs. 25%, + 12% [95% CI − 1.4% to + 25%], *p* = 0.08), compared to PMX-HA use in the full sample of the validation cohort (39% vs. 36%, + 2.9% [95% CI − 9.0% to + 15%], *p* = 0.70).

## Discussion

In this study, we statistically uncovered the heterogeneity in treatment effects of PMX-HA and identified a potential target phenotype of sepsis responsive to PMX-HA using a novel machine learning-based method (i.e., causal forest). Specifically, to streamline the use of PMX-HA on patients with sepsis, we estimated the individual treatment effects of PMX-HA on 28-day survival and identified patients with PT-INR > 1.4 or lactate > 3 mmol/L to be an optimal target of PMX-HA in the JSEPTIC-DIC study cohort. These derived criteria were validated in the EUPHRATES trial cohort which already used biomarker-guided enrichment with EAA.

Sepsis, defined as life-threatening organ dysfunction caused by a dysregulated host response to infection [26], is a heterogeneous syndrome. Seymour et al. identified four clinical phenotypes in sepsis, correlated with biomarkers and mortality [27]. Among these phenotypes, the *δ*-phenotype appears to have features consistent with disseminated intravascular coagulation (DIC), hyperlactatemia, liver dysfunction, and hypotension [28]. These characteristics align well with the optimal target of PMX-HA that we identified in this study, defined as patients with sepsis having coagulopathy and hyperlactatemia, underscoring the theoretical plausibility of the identified subpopulation. Endotoxin is one of the well-known triggers of DIC by activating both complements and cytokines [29], and thus is likely one of the molecular triggers for the pathophysiology of the *δ*-phenotype. Therefore, removal of circulating endotoxin by PMX-HA is expected to be effective in patients with coagulopathy caused by endothelial dysfunction and hyperlactatemia reflecting severe shock [3].

Identification of patients for any therapy combines clinical phenotyping with diagnostic testing. As PMX-HA targets endotoxin, the identification of endotoxemia using EAA is essential. However, high EAA without clinical evidence of organ failure for example using Multiple Organ Dysfunction Score (MODS) or SOFA score may not identify patients with sufficient disease burden to benefit from PMX-HA [8, 11]. Indeed, the EUPHRATES trial showed that PMX-HA was not effective on average in patients with MODS > 9 and EAA ≥ 0.60 [8], and its post-hoc analysis showed that patients with EAA between 0.60 and 0.90 may be the optimal target for the standard regimen of PMX-HA treatments [9, 10]. As a follow-up to these findings, an ongoing RCT (NCT03901807) is currently underway. The novelty of our findings is the potential efficacy of PMX-HA in patients with coagulopathy and hyperlactatemia. This suggests that targeting patients with these specific characteristics is more likely to result in positive outcomes with PMX-HA use. In other words, specific phenotyping using coagulopathy and/or hyperlactatemia (i.e., the *δ*-phenotype) in addition to endotoxemia may be superior to more general organ failure scores such as MODS and SOFA. Thus, it may be reasonable to consider initiating PMX-HA based on the patient's phenotype as well as EAA as investigated in the ongoing RCT.

## Limitations

Our study has limitations. First, because the cohorts used for our analysis were enrolled prior to the publication of the Sepsis-3 criteria [26] and the Surviving Sepsis Campaign Guidelines (SSCG) 2021 [30, 31] they might have been differently treated from current recommended guidelines. Therefore, our findings need to be evaluated with other cohorts or prospective studies, in which treatments are based on current treatment practice. Second, we developed the causal forest model using data from patients without RRT, which limits our ability to evaluate the importance of AKI, also a feature of the *δ*-phenotype, in selecting patients likely to respond to PMX-HA. However, based on our analysis using the validation cohort, our findings indicate that enrichment criteria are also valid in patients receiving RRT. Third, we have missing data in both the derivation and validation cohorts. This could have been a source of bias; however, we used a random forest imputation method—one of rigorous techniques for imputing both continuous and categorical variables at each frequency of missingness rates (5–40%) [16, 32]—for missing variables (0.4–30% of data in continuous variables) in the derivation cohort. We verified the derived results using the complete cases from the external RCT dataset (i.e., the EUPHRATES trial cohort). We believe that the influence of bias was minimized because the results from both cohorts were consistent. Fourth, limited clinical parameters on ICU admission (e.g., patient demographics, severity scores, laboratory data mainly related to coagulopathy) were used in the causal forest model development, which could cause unmeasured confounder bias. Also, the estimate of the treatment effects of PMX-HA in the derivation cohort could be affected by concomitant treatments, such as the use of antithrombin III and recombinant human soluble thrombomodulin (rhTM) (Additional file 1: Table S2). The validation of our findings in the EUPHRATES trial could strengthen the validity of the derived criteria for PMX-HA initiation, as the RCT design ensured a balanced comparison group, minimizing the impact of unmeasured confounders and concomitant treatments (e.g., anticoagulant use) on the outcomes. Finally, optimal timing and protocol of PMX-HA have not been determined in this study. In addition to the right targets of PMX-HA, these factors need to be investigated in the future study.

## Conclusions

The presence of coagulopathy and hyperlactatemia at ICU admission may be useful to select patients for PMX-HA; even those with high endotoxin activity. Our findings could help tailor the use of PMX-HA and serve to guide future trials.

### Supplementary Information

**Additional file 1**: Supplemental Tables and Figures.

## Data Availability

The data from the JSEPTIC-DIC study are publicly available (*Sci Data.* 2018;5:180243). The data from the EUPHRATES trial will not be shared beyond the requests from investigators of its trial.

## Abbreviations

PMX-HA: Polymyxin B hemadsorption
PMX-DHP: Polymyxin B direct hemoperfusion
RCT: Randomized controlled trial
EAA: Endotoxin assay activity
JSEPTIC-DIC: Japan septic disseminated intravascular coagulation
RRT: Renal replacement therapy
APACHE II: Acute physiology and chronic health evaluation II
SOFA: Sequential organ failure assessment
WBC: White blood cell
PT-INR: Prothrombin time-international normalized ratio
FDP: Fibrinogen/fibrin degradation products
ITEs: Individual treatment effects
HR: Hazard ratio
DIC: Disseminated intravascular coagulation
MODS: Multiple organ dysfunction score
