# Supplementary material for: Targeted therapy using polymyxin B hemadsorption in patients with sepsis: a post-hoc analysis of the JSEPTIC-DIC study and the EUPHRATES trial
Source: Crit Care. 2023 Jun 21;27:245. doi: 10.1186/s13054-023-04533-3 (PMC10286480; doi:10.1186/s13054-023-04533-3)
Supplement: Supplementary file 1 — Additional file 1: Supplemental Tables and Figures. [file 13054_2023_4533_MOESM1_ESM.docx]

**Supplementary information for:**

**Targeted therapy using Polymyxin B hemadsorption in patients with sepsis:**

**A post-hoc analysis of the JSEPTIC-DIC study and the EUPHRATES trial**

Itsuki Osawa, MD ^1^

Tadahiro Goto, MD, MPH, PhD ^2,3^

Daisuke Kudo, MD, PhD ^4^

Mineji Hayakawa, MD, PhD ^5^

Kazuma Yamakawa, MD, PhD ^6^

Shigeki Kushimoto, MD, PhD ^4^

Debra M Foster ^7^

John A Kellum, MD, MCCM ^7,8^

Kent Doi, MD, PhD ^1^

**Affiliations:**

1. Department of Emergency and Critical Care Medicine, The University of Tokyo Hospital, Tokyo, Japan.
2. Department of Clinical Epidemiology and Health Economics, School of Public Health, The University of Tokyo, Tokyo, Japan.
3. TXP Medical Co. Ltd., Tokyo, Japan.
4. Division of Emergency and Critical Care Medicine, Tohoku University Graduate School of Medicine, Miyagi, Japan.
5. Department of Emergency Medicine, Hokkaido University Hospital, Hokkaido, Japan.
6. Department of Emergency and Critical Care Medicine, Osaka Medical and Pharmaceutical University, Osaka, Japan.
7. Spectral Medical, Toronto, Ontario, Canada.
8. Center for Critical Care Nephrology, Department of Critical Care Medicine, University of Pittsburgh, PA, USA.

Supplementary information content

[Supplemental Table 1. The rate of missingness of covariates in the derivation (the JSEPTIC-DIC study) cohort 3](#_Toc135994859)

[Supplemental Table 2. Baseline characteristics of patients in the derivation (the JSEPTIC-DIC study) cohort 4](#_Toc135994860)

[Supplemental Table 3. Baseline characteristics of patients in the validation (the EUPHRATES trial) cohort 5](#_Toc135994861)

[Supplemental Table 4. Patient characteristics of PMX-HA targeted patients in the derivation (the JSEPTIC-DIC study) cohort 6](#_Toc135994862)

[Supplemental Table 5. Patient characteristics of PMX-HA targeted patients in the validation (the EUPHRATES trial) cohort 7](#_Toc135994863)

[Supplemental Figure 1. Analytic workflow of our analyses 8](#_Toc135994864)

[Supplemental Figure 2. Predicted treatment effects of PMX-HA on 28-day survival across the levels of four candidate determinants of treatment effects 9](#_Toc135994865)

[Supplemental Figure 3. Identification of the potential PMX-HA indication using the policytree algorithm 10](#_Toc135994866)

# **Supplemental Table 1. The rate of missingness of covariates in the derivation (the JSEPTIC-DIC study) cohort**

| Characteristic | Derivation cohort  (n = 1,911) |
| --- | --- |
|  | Missing, n (%) |
| Patient demographics |  |
| Age | 0 (0) |
| Female sex | 0 (0) |
| Patient severity |  |
| APACHE II score | 1 (0.0) |
| SOFA score | 2 (0.0) |
| Respiratory | 2 (0.0) |
| Coagulation | 0 (0) |
| Liver | 0 (0) |
| Cardiovascular | 0 (0) |
| Central nervous system | 0 (0) |
| Renal | 0 (0) |
| Laboratory tests |  |
| White blood cells | 7 (0.4) |
| Platelet | 7 (0.4) |
| PT-INR | 116 (6) |
| Fibrinogen | 419 (22) |
| FDP | 577 (30) |
| Lactate | 285 (15) |

Abbreviations: APACHE II score = Acute Physiology and Chronic Health Enquiry II score, SOFA score = Sequential Organ Failure Assessment score, PT-INR = Prothrombin Time and International Normalized Ratio, FDP = Fibrin Degradation Products

# **Supplemental Table 2. Baseline characteristics of patients in the derivation (the JSEPTIC-DIC study) cohort**

| Characteristics | PMX-HA use  (n = 350) | Control  (n = 1,561) |
| --- | --- | --- |
| Patient demographics |  |  |
| Age | 72 (62 - 80) | 74 (63 - 82) |
| Female sex, n (%) | 164 (47%) | 653 (42%) |
| Infection site, n (%) |  |  |
| Lung | 28 (8%) | 461 (30%) |
| Abdominal | 238 (68%) | 404 (26%) |
| Others | 84 (24%) | 696 (44%) |
| Blood culture positive, n (%) | 255 (73%) | 1,175 (75%) |
| Patient severity |  |  |
| APACHE II score | 21 (16 - 27) | 21 (16 - 27) |
| SOFA score | 9 (7 - 12) | 8 (6 - 11) |
| Respiratory | 2 (1 - 3) | 2 (1 - 3) |
| Coagulation | 1 (0 - 2) | 1 (0 - 2) |
| Liver | 0 (0 - 1) | 0 (0 - 1) |
| Cardiovascular | 3 (2 - 4) | 2 (0 - 3) |
| Central nervous system | 1 (0 - 2) | 1 (0 - 3) |
| Renal | 1 (0 - 2) | 1 (0 - 2) |
| Laboratory tests |  |  |
| White blood cells [x10^3^/μL] | 6 (2 - 16) | 12 (6 - 18) |
| Platelet [x10^4^/μL] | 12 (7 – 18) | 14 (8 - 22) |
| PT-INR | 1.47 (1.26 - 1.72) | 1.29 (1.15 - 1.49) |
| Fibrinogen [mg/dL] | 346 (214 - 469) | 430 (324 - 541) |
| FDP [μg/mL] | 23 (12 - 42) | 22 (13 - 37) |
| Lactate [mmol/L] | 3.9 (2.4 - 6.4) | 2.8 (1.8 – 5.0) |
| Concomitant treatments |  |  |
| Antithrombin III use | 167 (48%) | 400 (26%) |
| rhTM use | 113 (32%) | 341 (22%) |
| Heparin use | 29 (8.3%) | 170 (11%) |
| Outcome |  |  |
| 28-day mortality, n (%) | 53 (15%) | 286 (18%) |

Values represent median (IQR), unless otherwise indicated.

Abbreviations: PMX-HA = Polymyxin B Hemadsorption, APACHE II score = Acute Physiology and Chronic Health Enquiry II score, SOFA score = Sequential Organ Failure Assessment score, PT-INR = Prothrombin Time and International Normalized Ratio, FDP = Fibrinogen/Fibrin Degradation Products, rhTM = recombinant human soluble thrombomodulin

# **Supplemental Table 3. Baseline characteristics of patients in the validation (the EUPHRATES trial) cohort**

| Characteristic | PMX-HA use  (n = 132) | Control  (n = 154) |
| --- | --- | --- |
| Patient demographics |  |  |
| Age | 62 (52 - 70) | 58 (48 - 69) |
| Female sex, n (%) | 54 (41%) | 61 (40%) |
| Infection site, n (%) |  |  |
| Lung | 39 (30%) | 62 (40%) |
| Abdominal | 35 (27%) | 38 (25%) |
| Others | 58 (44%) | 54 (35%) |
| Blood culture positive, n (%) | 47 (36%) | 43 (28%) |
| Patient severity |  |  |
| APACHE II score | 30 (25 - 34) | 29 (23 - 34) |
| SOFA score | 12 (10 - 14) | 12 (11 - 14) |
| MODS | 10 (8 – 12) | 10 (8 – 12) |
| EAA | 0.79 (0.69 – 0.87) | 0.75 (0.66 – 0.85) |
| Kidney status |  |  |
| AKIN stage |  |  |
| Stage 0 | 35 (27%) | 44 (29%) |
| Stage 1 | 22 (17%) | 20 (13%) |
| Stage 2  S | 17 (13%) | 20 (13%) |
| Stage 3 | 58 (44%) | 70 (45%) |
| RRT at randomization | 23 (17%) | 39 (25%) |
| Laboratory tests |  |  |
| White blood cells [x10^3^/μL] | 14 (8 - 24) | 14 (7 - 21) |
| Platelet [x10^4^/μL] | 12 (7 – 19) | 12 (8 - 21) |
| PT-INR | 1.50 (1.31 - 1.79) | 1.47 (1.22 - 1.75) |
| Lactate [mmol/L] | 2.9 (1.7 – 5.0) | 2.8 (1.6 – 5.3) |
| Outcome |  |  |
| Renal replacement therapy over 28 days | 68 (52%) | 78 (51%) |
| 28-day mortality, n (%) | 40 (30%) | 58 (38%) |

Values represent median (IQR), unless otherwise indicated.

Abbreviations: PMX-HA = Polymyxin B Hemadsorption, APACHE II score = Acute Physiology and Chronic Health Enquiry II score, SOFA score = Sequential Organ Failure Assessment score, MODS = Multiple Organ Dysfunction Score, EAA = Endotoxin Activity Assay, AKIN stage = Acute Kidney Injury Network stage, PT-INR = Prothrombin Time and International Normalized Ratio, FDP = Fibrinogen/Fibrin Degradation Products

# **Supplemental Table 4. Patient characteristics of PMX-HA targeted patients in the derivation (the JSEPTIC-DIC study) cohort**

| Characteristic | Targeted population  (n = 1,203) | Non-targeted population  (n = 708) |
| --- | --- | --- |
| Patient demographics |  |  |
| Age | 74 (63 - 82) | 72 (62 - 80) |
| Female sex, n (%) | 519 (43%) | 298 (58%) |
| Infection site, n (%) |  |  |
| Lung | 275 (23%) | 214 (30%) |
| Abdominal | 464 (39%) | 178 (25%) |
| Others | 464 (39%) | 316 (45%) |
| Blood culture positive, n (%) | 911 (76%) | 519 (73%) |
| Patient severity |  |  |
| APACHE II score | 23 (17 - 28) | 18 (14 - 23) |
| SOFA score | 9 (7 - 12) | 7 (4 - 9) |
| Respiratory | 2 (1 - 3) | 2 (1 - 3) |
| Coagulation | 1 (0 - 2) | 0 (0 - 2) |
| Liver | 0 (0 - 1) | 0 (0 - 1) |
| Cardiovascular | 3 (1 - 4) | 1 (0 - 3) |
| Central nervous system | 1 (1 - 3) | 1 (0 - 2) |
| Renal | 1 (0 - 2) | 0 (0 - 1) |
| Laboratory tests |  |  |
| White blood cells [x10^3^/μL] | 11 (3 - 18) | 12 (7 - 19) |
| Platelet [x10^4^/μL] | 13 (7 – 19) | 15 (9 - 24) |
| PT-INR | 1.46 (1.25 - 1.70) | 1.20 (1.10 - 1.29) |
| Fibrinogen [mg/dL] | 378 (262 - 485) | 481 (393 - 589) |
| FDP [μg/mL] | 26 (13 - 47) | 20 (12 - 28) |
| Lactate [mmol/L] | 4.4 (3.2 - 6.8) | 1.8 (1.2 – 2.3) |
| Outcome |  |  |
| 28-day mortality, n (%) | 275 (23%) | 64 (9%) |

Values represent median (IQR), unless otherwise indicated.

Abbreviations: PMX-HA = Polymyxin B Hemadsorption, APACHE II score = Acute Physiology and Chronic Health Enquiry II score, SOFA score = Sequential Organ Failure Assessment score, PT-INR = Prothrombin Time and International Normalized Ratio, FDP = Fibrinogen/Fibrin Degradation Products

# **Supplemental Table 5. Patient characteristics of PMX-HA targeted patients in the validation (the EUPHRATES trial) cohort**

| Characteristic | Targeted population  (n = 214) | Not-targeted population  (n = 72) |
| --- | --- | --- |
| Patient demographics |  |  |
| Age | 61 (49 - 71) | 58 (51 - 66) |
| Female sex, n (%) | 85 (40%) | 30 (42%) |
| Infection site, n (%) |  |  |
| Lung | 75 (35%) | 26 (36%) |
| Abdominal | 13 (18%) | 13 (18%) |
| Others | 126 (59%) | 33 (46%) |
| Blood culture positive, n (%) | 75 (35%) | 15 (21%) |
| Patient severity |  |  |
| APACHE II score | 30 (25 - 35) | 27 (20 - 32) |
| SOFA score | 13 (11 - 14) | 12 (10 - 13) |
| MODS | 10 (9 – 13) | 10 (7 – 11) |
| EAA | 0.78 (0.67 – 0.87) | 0.74 (0.65 – 0.83) |
| EAA of 0.6-0.9, n (%) | 177 (83%) | 63 (88%) |
| EAA of >0.9, n (%) | 37 (17%) | 9 (12%) |
| Kidney status |  |  |
| AKIN stage |  |  |
| Stage 0 | 27 (38%) | 52 (24%) |
| Stage 1 | 8 (11%) | 34 (16%) |
| Stage 2  S | 12 (17%) | 25 (12%) |
| Stage 3 | 25 (35%) | 103 (48%) |
| RRT at randomization | 8 (11%) | 54 (25%) |
| Laboratory tests |  |  |
| White blood cells [x10^3^/μL] | 14 (7 - 23) | 13 (8 - 18) |
| Platelet [x10^4^/μL] | 14 (10 – 22) | 11 (6 - 19) |
| PT-INR | 1.59 (1.44 - 1.89) | 1.17 (1.04 - 1.28) |
| Lactate [mmol/L] | 3.8 (2.3 – 6.4) | 1.4 (1.1 – 2.1) |
| Outcome |  |  |
| Renal replacement therapy over 28 days | 120 (56%) | 26 (36%) |
| 28-day mortality, n (%) | 86 (40%) | 12 (17%) |

Values represent median (IQR), unless otherwise indicated.

Abbreviations: PMX-HA = Polymyxin B Hemadsorption, APACHE II score = Acute Physiology and Chronic Health Enquiry II score, SOFA score = Sequential Organ Failure Assessment score, MODS = Multiple Organ Dysfunction Score, EAA = Endotoxin Activity Assay, AKIN stage = Acute Kidney Injury Network stage, PT-INR = Prothrombin Time and International Normalized Ratio, FDP = Fibrinogen/Fibrin Degradation Products

# **Supplemental Figure 1. Analytic workflow of our analyses**

Abbreviations: PMX-HA = Polymyxin B Hemadsorption

# **Supplemental Figure 2. Predicted treatment effects of PMX-HA on 28-day survival across the levels of four candidate determinants of treatment effects**

The heatmaps indicate the predicted therapeutic effect of PMX-HA on 28-day survival while changing the levels of APACHE II score, platelet, PT-INR, and lactate based on the developed causal forest model. The closer the color is to orange, the more effective PMX-HA is; the closer the color is to blue, the less effective PMX-HA is.

Abbreviations: PMX-HA = Polymyxin B Hemadsorption, APACHE II score = Acute Physiology and Chronic Health Enquiry II score, PT-INR = Prothrombin Time and International Normalized Ratio

# **Supplemental Figure 3. Identification of the potential PMX-HA indication using the policytree algorithm**

Applying the policytree algorithm to the derivation cohort, we attempted to determine the potential indication of PMX-HA using the candidate determinants of treatment effects (i.e., APACHE II score, platelet count, PT-INR, and lactate). We show a tree indicating optimal targets of PMX-HA. “Leaf node action = 1” refers to patients for whom PMX-HA is not recommended, and “leaf node action = 2” refers to patients for whom PMX-HA is recommended. The policytree algorithm showed that identifying the optimal target of PMX-HA using a combination of PT-INR and lactate could be useful. We identified patients with PT-INR > 1.43 or (PT-INR ≤ 1.43 and lactate > 3.22 mmol/L) as a potential target of PMX-HA. For simplicity, we defined the optimal target as patients with PT-INR > 1.4 or lactate > 3 mmol/L.

Abbreviations: PMX-HA = Polymyxin B Hemadsorption, PT-INR = Prothrombin Time and International Normalized Ratio, Lac = Lactate
